# Advancing Diagnostic Safety Research: Results of a Systematic Research Priority Setting Exercise

**DOI:** 10.1007/s11606-020-06428-3

**Published:** 2021-02-09

**Authors:** Laura Zwaan, Robert El-Kareh, Ashley N. D. Meyer, Jacky Hooftman, Hardeep Singh

**Affiliations:** 1 Erasmus Medical Center Rotterdam, Institute of Medical Education Research Rotterdam, Rotterdam, The Netherlands; 2 Department of Medicine, University of California at San Diego, San Diego, CA USA; 3 Center for Innovations in Quality, Effectiveness and Safety, Michael E. DeBakey VA Medical Center, Houston, TX USA; 4 Baylor College of Medicine, Houston, TX USA

**Keywords:** diagnostic safety, patient safety, research priorities, medical error

## Abstract

**Background:**

Diagnostic errors are a major source of preventable harm but the science of reducing them remains underdeveloped.

**Objective:**

To identify and prioritize research questions to advance the field of diagnostic safety in the next 5 years.

**Participants:**

Ninety-seven researchers and 42 stakeholders were involved in the identification of the research priorities.

**Design:**

We used systematic prioritization methods based on the Child Health and Nutrition Research Initiative (CHNRI) methodology. We first invited a large international group of expert researchers in various disciplines to submit research questions while considering five prioritization criteria: (1) usefulness, (2) answerability, (3) effectiveness, (4) potential for translation, and (5) maximal potential for effect on diagnostic safety. After consolidation, these questions were prioritized at an in-person expert meeting in April 2019. Top-ranked questions were subsequently reprioritized through scoring on the five prioritization criteria using an online questionnaire. We also invited non-research stakeholders to assign weights to the five criteria and then used these weights to adjust the final prioritization score for each question.

**Key Results:**

Of the 207 invited researchers, 97 researchers responded and 78 submitted 333 research questions which were then consolidated. Expert meeting participants (*n* = 21) discussed questions in different breakout sessions and prioritized 50, which were subsequently reduced to the top 20 using the online questionnaire. The top 20 questions addressed mostly system factors (e.g., implementation and evaluation of information technologies), teamwork factors (e.g., role of nurses and other health professionals in the diagnostic process), and strategies to engage patients in the diagnostic process.

**Conclusions:**

Top research priorities for advancing diagnostic safety in the short-term include strengthening systems and teams and engaging patients to support diagnosis. High-priority areas identified using these systematic methods can inform an actionable research agenda for reducing preventable diagnostic harm.

**Supplementary Information:**

The online version contains supplementary material available at 10.1007/s11606-020-06428-3.

## INTRODUCTION

High-quality research is essential to accelerate quality and safety of healthcare.^1^ One emerging risk area is diagnostic error, with estimates that at least 1 in 20 adults will have a diagnostic error annually in the outpatient setting.^2^ In hospitals, diagnostic error could involve 0.7% of adult hospitalizations and 5.6% of medical 7-day readmissions and many result in serious patient harm.^3–5^ However, substantial research gaps remain that limit diagnostic error reduction.^6–8^ This is not surprising because the diagnostic process is inherently complex and involves decision-making under uncertain conditions and limited time.^9–11^

The science of reducing diagnostic error remains underdeveloped and requires newer approaches, especially because the current medical research funding largely adopts a disease-focused approach, whereas the diagnostic process cuts across thousands of diseases.^12, 13^ Potential research opportunities can be broadly classified into three areas: error epidemiology, contributory factors, and interventions.^14^ The National Academies of Sciences, Engineering, and Medicine’s (NASEM) report *Improving Diagnosis in Health Care*^15^ defined diagnostic error as the failure to (a) establish an accurate and timely explanation of the patient’s health problem(s) or (b) communicate that explanation to the patient. It concluded that that there is an urgent need for research on the diagnostic process and diagnostic errors and called for “a coordinated federal research agenda, committed funding, and significant public–private collaborations to enhance research in this critical area.” The NASEM report included a list of potential research areas but prioritization of research areas and development of a specific set of actionable research questions to create impact on practice and patient care was considered outside the scope of the report. Efforts are needed to inform a deeper understanding of how to reduce missed opportunities in diagnosis and achieve correct and timely diagnosis while maximizing patient experiences. These efforts include identifying the main failure points given that diagnosis evolves over time within a complex sociotechnical health system,^16, 17^ and developing, implementing, and testing specific interventions in order to achieve diagnostic excellence.^18^

In the past decade, few researchers have embarked on projects to advance the scientific understanding of diagnostic error and even fewer are using multidisciplinary perspectives to address evidence gaps.^14, 19–22^ Much of this research is fragmented with little assurance it focuses on the right questions.^23^ Additional insights are needed to identify the most urgent and impactful questions to promote research that is more actionable and reduces patient harm. Therefore, we conducted a systematic research priority-setting exercise to identify and prioritize research questions to advance the field of diagnostic safety.

## METHODS

### Overview

We used established systematic research prioritization methods based on the Child Health and Nutrition Research Initiative (CHNRI).^24^ The CHNRI is a systematic and well-established method to identify research priorities.^25–27^ We invited an international group of expert researchers in several disciplines related to diagnostic error to submit research questions. The submitted questions were prioritized using predefined prioritization criteria ensuring a transparent and systematic priority-setting process to minimize potential bias.

### Scope and Prioritization Criteria

As a first step, the core research group (LZ, REK, AM, HS) defined the project scope as topics that would advance the field of diagnostic safety in the next 3–5 years in order to reduce patient harm in the diagnosis process. We also selected and adapted the prioritization criteria for developing and evaluating the research questions from the entire list of criteria described in the CHNRI approach (see Text Box 1).^28^ Diagnostic safety was defined as the prevention of errors and adverse effects to patients associated with the diagnostic process. We used the NASEM conceptualization of the diagnostic process.^15^ We limited the scope by deeming screening decisions of asymptomatic patients as outside the focus of the project. However, research questions related to diagnostic evaluation of abnormal screening results were within scope. Treatment decisions were only included if they were relevant to the diagnostic process.

**Text Box 1**. Prioritization criteria

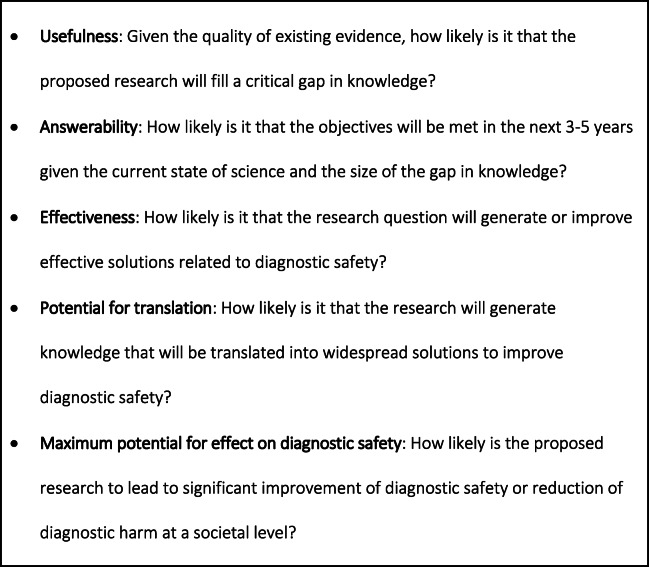



### Expert Selection

To ensure a broad sample of researchers, we searched for researchers from diverse backgrounds active in a variety of research fields with expertise relevant to diagnostic safety. The search included the following: (1) authors of key diagnostic safety articles in PubMed, Google Scholar, and AHRQ PSNet; (2) NASEM “Improving Diagnosis in Health Care” report committee members, reviewers, and authors frequently cited in the report;^15^ (3) members of research committees of various societies (e.g., Society of General Internal Medicine; Society of Medical Decision Making; Human Factors and Ergonomics Society); and (4) recipients of quality and safety-related grants and awards.

### Soliciting Research Questions in Round 1

We invited the researchers via an email and requested they submit research questions considering the project scope and the five prioritization criteria. If researchers declined participation, we requested a brief reason. If they agreed to participate, they entered the questions as free text in an online questionnaire in Qualtrics Research Suite (Qualtrics, Provo, UT). We asked them to indicate the domain of each question: (1) measuring burden of the problem, (2) identifying contributing factors, (3) developing and testing effectiveness of solutions, and (4) other for miscellaneous questions.

### Expert Meeting

The list of research questions was initially prioritized at an in-person 1.5-day expert meeting composed of international experts in April 2019 to select high-priority questions (for a list of participants see supplementary material). The group consisted of researchers with expertise in diagnostic safety research as well as related fields such as human factors, informatics, and social sciences. We also included a patient representative and representatives of funders.

Prior to the meeting, the core research group checked the quality of questionnaire responses, removed responses outside the scope and duplicate questions, and merged similar questions. Furthermore, all questions submitted to the “other” domain were recategorized, making this domain obsolete.

**Figure 1:**
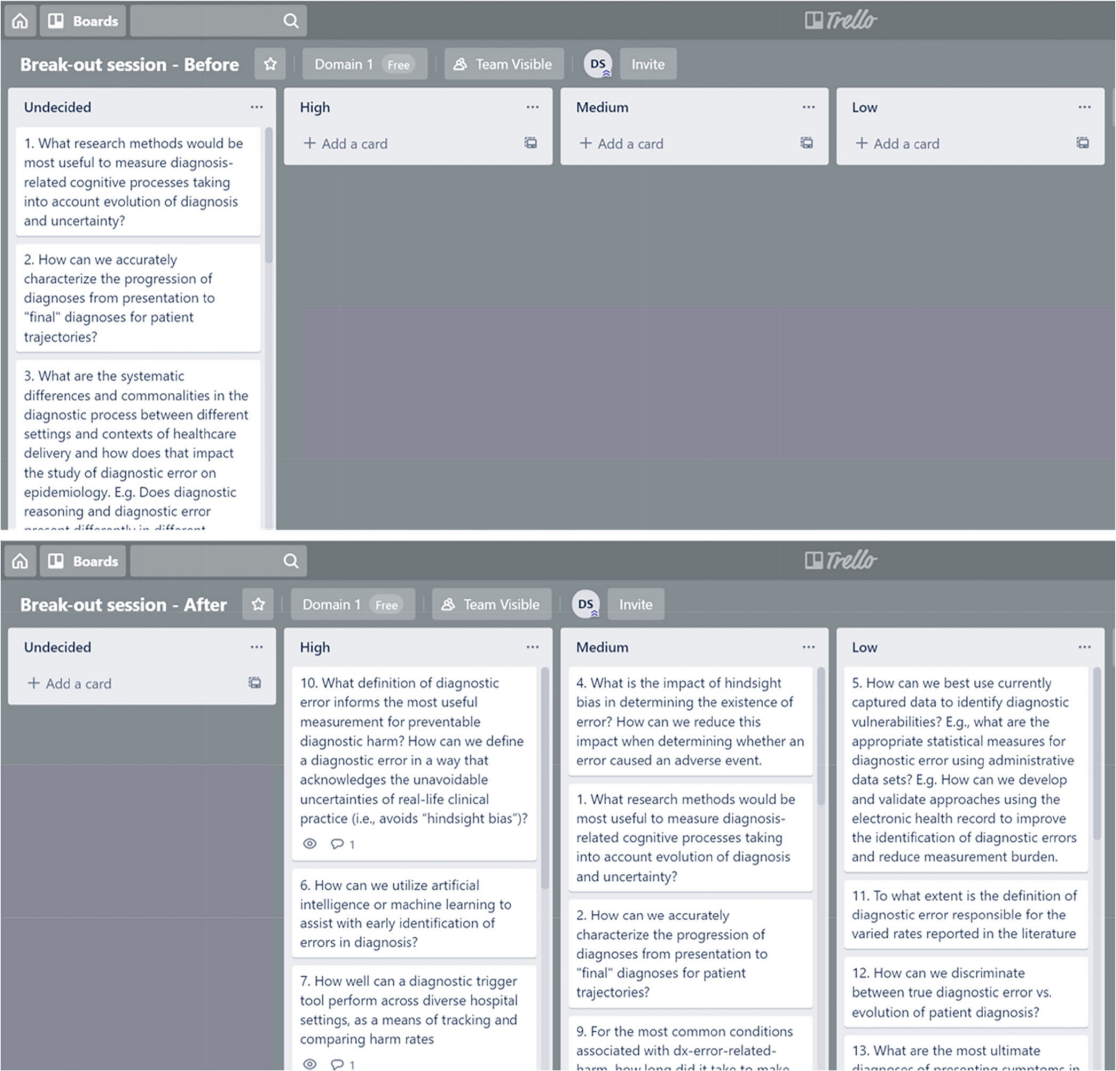
An example of a Trello board. The top board is an example of the state of a Trello board before discussion; all questions appear in the “Undecided” column. The bottom board is an example of the state of a Trello board after discussion; each question is moved to the High, Medium, or Low Priority column.

During the expert meeting, we conducted a series of prioritization exercises for each of the three domains. The intent was to identify the top 15 research questions in each domain for a total of 45 research questions. We used Trello (Trello by Atlassian, New York City, NY), a free online program for organizing lists and “cards” on a virtual whiteboard (see Fig. 1).

The large group was divided into three equal breakout groups for each of the three domain prioritization exercises (i.e., one each for burden, contributory factors, and interventions) keeping a similar multidisciplinary representation that was present in the larger group. The virtual whiteboards (Fig. 1, top board) initially contained four lists: Undecided (i.e., all of the research questions for that session that needed to be sorted into the other three lists), and High, Medium, and Low Priority. Each group projected their own version of the whiteboard on a screen, discussed each question, and moved the questions from undecided to one of the priority levels (Fig. 1, bottom board). At the end of the breakout, all participants came together for a plenary discussion. At the start of the plenary, responses from the three groups were combined and questions that made the high-priority list in at least two groups automatically moved to the final high-priority list. The entire group then discussed the remaining questions and assigned them to high, medium, or low priority. This process was followed for all three domains.

### Stakeholders

To recognize the important role of non-researcher stakeholders in the field of diagnostic safety, we invited them to weigh the prioritization criteria in order of importance. The weights would affect the final prioritization score. We selected various types of stakeholders based on their engagement, work, or expertise relevant to diagnosis and invited stakeholder representatives from the following categories: risk managers/patient safety professionals, patient advocates, clinicians, liability insurers, funders, educators, policy makers, health system leaders, developers of decision support systems, and patient safety organizations.

The stakeholders received an email from the study principal investigators with a request to indicate how important they deemed each of the prioritization criteria. If they agreed to participate, they reviewed the prioritization criteria and ranked them in order of importance (“1” most important–“5” least important) through a short questionnaire in the Qualtrics Research Suite. The weights were determined by calculating the average importance score for each criterion and then dividing the average expected score of 3.0 (i.e., the average expected rank if all criteria were valued the same) by the average score.^29^ Prioritization criteria with a weight < 1.0 would have a lower contribution in determining the final priority score whereas criteria with a weight > 1.0 would have a higher contribution in the determination of the priority score.

### Final Prioritization in Round 2

All researchers who submitted questions were invited to score the high-priority questions that resulted from the expert panel using the five predefined prioritization criteria. Researchers indicated whether the question met the criteria (100 points assigned), whether the question did not meet the criteria (0 points assigned), or whether they were undecided whether the criteria was met (50 points assigned). Recognizing that researchers would have unique expertise that may not cut across all the diverse areas and disciplines, we requested them to score only the areas they were comfortable with. The scores of the researcher resulted in a priority score for each of the research questions.^30^ To obtain the final weighted Research Prioritization Score (WRPS), the score for each of the criteria was multiplied by the weight assigned to that criterion by the stakeholders.

To obtain insights into the agreement between the researchers who scored the research questions on the prioritization criteria, we used the Average Expert Agreement (AEA) score.^30^ This score represents the average proportion of experts who agreed on the responses for the five prioritization criteria per question and a valuable indicator of the agreement between experts on the importance of the research question.

### Ethical Approval

Ethical approval was reviewed by the Medical Ethics Research Committee of Erasmus Medical Center, Rotterdam, The Netherlands.

## RESULTS

Of the 207 invited researchers, 97 researchers responded (46.9% response rate). Of this group, 78 researchers from over 10 different countries submitted research questions and 19 declined participation, for lack of time or because they felt they had insufficient expertise in diagnostic safety. The sample represented fields of quality/patient safety, human factors, social science, implementation research, health services research, health IT/informatics, and medical decision making/clinical reasoning.

Researchers submitted 333 research questions in round 1: 77 for measuring burden, 89 for contributing factors, 145 for interventions, and 22 for the other category. Prior to the expert meeting, the core research group consolidated the questions down to 177 questions. This reduction was mainly because of a large number of duplicates and merging of similar questions, but a few questions out of the scope of the project were excluded.

During the expert meeting, participants (*n* = 21) discussed all 177 questions in three different sessions for burden, contributory factors, and interventions respectively. Each session was further divided into three breakout groups that discussed the same questions. The breakout group discussion prioritized 10, 10, and 9 questions immediately to a high-priority list for burden, contributory factors, and interventions respectively. In the plenary group discussions, the remaining questions were discussed resulting in a final high-priority list of 51 questions, 13 questions for measuring burden, 17 questions for contributing factors, and 20 questions for interventions.

Of the 76 invited stakeholders, 43 people responded (response rate 56.6%). Of those stakeholders, 42 submitted weights for the prioritization criteria and one person did not consider him/herself a stakeholder for the field. The stakeholders had assigned the weights (1 being a neutral weight). Maximum potential for effect on diagnostic safety = 1.30; effectiveness = 1.11; potential for translation = 1.06; usefulness = 0.95; and answerability = 0.75.

Of the 78 invited researchers who submitted questions on round 1, 49 responded (response rate 62.8%) in the reprioritization exercise in round 2. On average, the researchers indicated 5.7 (11.4%) questions as outside their expertise. The weighted top 20 questions are listed in Table 1. Specifically, the WRPS are listed as well as each of the prioritization criteria and the AEA score.

**Table 1:** Top 20 Prioritized Research Questions Including Weighted Research Priority Scores, Criterion Scores, and Average Expert Agreement (AEA)

	Proposed research question	Weighted RPS	Domain	Usefulness	Answerability	Effectiveness	Potential for translation	Effect on diagnostic safety	AEA*
1	How do we better develop the evidence base of diagnostic decision support tools (e.g., differential diagnosis generators, decision support for test selection and interpretation, etc.) in terms of effectiveness and implementation? i.e., how can we effectively integrate diagnostic decision support into clinician and patient workflows?	90.00	Technology	94	81	89	90	82	0.81
2	How can EHRs and patient portals be optimized (through local preferences or EHR vendor changes) to most effectively manage abnormal test results, such as incidental findings or test results that come back after transitions of care (e.g., discharge from ED or hospital)?	88.71	Technology	94	89	84	89	77	0.79
3	What are effective strategies to include nurses and other health professionals in optimizing the diagnostic process and identifying and preventing potential harmful diagnostic situations?	88.42	Teamwork	95	88	86	83	79	0.77
4	How can we best bring expert knowledge about diagnostic test selection and result interpretation to ordering providers at the point of care?	88.42	Teamwork	93	86	89	83	79	0.79
5	How do different forms of health IT and associated information content, information displays and health IT-human interactions impact clinical decision-making and the diagnostic process? Different forms of health IT include EHRs, telehealth, portals, apps. Information content broadly includes decision support, use of coded data and documentation. Information displays includes all types of visualization modalities. Different forms of interactions could include clinician-patient interactions affected by computers, use or scribes.	86.13	Technology	92	83	81	79	83	0.74
6	How do we develop and evaluate performance of diagnostic trigger tools that can be used to identify or prevent diagnostic errors across the care continuum?	85.95	Measurement	91	84	81	84	78	0.71
7	How can systematic feedback be given to providers in different settings/specialties to improve metacognition (including calibration between confidence and accuracy) and improve diagnostic processes and outcomes without increasing over-testing and overdiagnosis?	85.42	Cognition	96	84	79	82	77	0.76
8	How do work system factors such as workload (and work compression) time-pressure and interruptions affect the frequency and types of diagnostic errors?	85.33	Epidemiology	91	82	82	75	84	0.76
9	What types of EHR design and functionality can effectively and efficiently summarize important historical patient context and new clinical findings to facilitate the making of an otherwise unrecognized diagnosis?	83.76	Technology	86	75	81	80	81	0.70
10	Understand how AI can be used effectively to augment diagnostic decision-making, including probabilistic decision-making; identify which AI-based tools and techniques are useful to improve diagnostic accuracy and how AI can be best integrated into the clinician's diagnostic process-related workflow.	83.60	Technology	90	83	79	80	75	0.69
11	What are the effective strategies in which to include patients, families and caregivers in preventing diagnostic errors (e.g., by using patient feedback to increase learning and to create safety nets)?	83.14	Teamwork	88	79	83	78	76	0.68
12	What are the barriers and enablers to effective diagnostic teamwork observed in various situations (e.g., by practice settings, different diagnostic time courses, different team configurations, etc.). How can we leverage methods and theories from cognitive psychology and human factors to examine and support effective teamwork?	82.88	Teamwork	85	76	78	81	80	0.71
13	How do we best use patient input and feedback to identify diagnostic error in a reliable and valid fashion?	82.79	Teamwork	94	79	80	79	72	0.70
14	In what conditions can team-based approaches to diagnosis (such as use of collective intelligence or other methods leveraging distributed models of cognition especially through use of technology), significantly increase diagnostic accuracy in real world clinical settings?	81.54	Teamwork	95	76	81	76	70	0.67
15	How can we use IT-based tools and techniques to better capture, analyze, visualize, represent and share clinical decision making related to the diagnostic process? These would include decision-making processes related to uncertainty, watchful waiting, differential diagnosis, Bayesian reasoning.	80.77	Technology	87	67	79	77	78	0.67
16	What are the most effective methods to leverage existing electronic data to do real time (or quasi “real time”, meaning a clinically meaningful timeframe) measurement of diagnostic error? Provide actionable feedback of diagnostic accuracy at the individual clinician level in “real time”?	80.73	Measurement	91	78	78	74	72	0.66
17	Diagnostic accuracy/expertise depends on experiential knowledge—what are the most effective strategies in medical education for improving experiential knowledge prior to independent practice? Can we jump start the acquisition of experience via simulated diagnostic experiences?	80.00	Cognition	87	79	76	81	67	0.65
18	Can we improve diagnostic safety by facilitating shared decision making in the diagnostic process, i.e., by discussing the risks and benefits of watchful waiting vs. additional diagnostic testing and treatment options?	79.89	Cognition	92	74	78	76	69	0.66
19	How can we effectively use near real time second review considering factors such as case selection (random or systematic), specialty (within specialty or multidisciplinary) to impact calibration, knowledge, and error reduction?	78.91	Measurement	84	85	76	69	72	0.68
20	Are diagnostic errors more or less likely in specific patient population? For example, certain demographics (race/ethnicity), certain socioeconomic or social determinants of health factors or other factors (prison, homelessness, migrant etc.) may lead to disparities with respect to diagnostic delays and errors.	78.81	Epidemiology	87	85	72	77	66	0.65

*The AEA is the Average Expert Agreement, where 0 means there is no agreement and 1 represents full agreement. The closer the number is to 1, the more the experts agreed on the prioritization scores for the question

## DISCUSSION

Using systematic, transparent, and objective methods that included input from a large group of researchers and stakeholders, we identified a list of top 20 research questions that inform a research agenda that could be supported by several types of funding agencies. Answers to these questions can identify high-risk areas and key underlying causes of diagnostic errors for which promising interventions can be developed and tested. Our findings can guide research funders on development of future requests for proposals as well as encourage researchers to think about specific research ideas, hypothesis, and specific aims within the broader context of the research priorities we identified.

Because we solicited research questions for a 3–5-year horizon, unsurprisingly, 6 of the top 10 research priorities were from the intervention category. Reflecting the current state of unanswered questions^31^ and challenges that have emerged with use of information technology,^32, 33^ technology-related questions emerged prominently in the top 10. These questions focused on improving diagnostic decision support systems, improving design and functionality of electronic health records specifically for diagnosis and use of artificial intelligence in the diagnostic process. The higher priority scores to interventions were thus in line with the scope of the project aiming for harm reduction in 3–5 years. This also reflects the higher weight that stakeholders gave to the evaluation criterion “Maximum Potential for Effect on Diagnostic Safety.” Strategies to engage patients in the diagnostic process and reducing error also emerged prominently and so did research on teamwork, consistent with other areas of patient safety.^34, 35^

Emphasis on teamwork is especially relevant given that diagnosis is a team sport that involves several members including the frontline clinicians, patients, nurses, laboratory/pathology, and radiology.

Our project used a collaborative interdisciplinary approach to develop questions rather than individual disciplines coming up with their own priorities for reducing diagnostic error. This approach is consistent with the team sport nature of diagnosis and likely led to different results than what may have been seen if each member group had worked in silos. For instance, addressing test ordering and selection problems involves not just frontline clinicians such as emergency, hospital, and general adult and pediatric medicine but also laboratorians and radiologists. Each should have input for research questions. As a result, while several testing-related questions that crossed disciplines emerged in the top 20, more specific and individual research priorities, such as within the laboratory or radiology fields, did not. Another advantage of using this discipline-agnostic approach was emergence of novel ideas pertinent to several aspects of medicine, for instance how to deliver feedback to clinicians about their diagnostic performance. A collaborative approach across researchers from multiple disciplines thus enhanced the quality of the research questions.

Questions related to measurement of burden and contributing factors did not emerge to the top, even though this could be considered as a required concurrent or initial step to examining the effectiveness of interventions. For instance, several systemic factors need to be addressed in order to make changes including legal and regulatory issues, malpractice-related concerns, and defensive medicine, developing a safety culture and overcoming frontline implementation challenges to solve quality and safety problems. All of these “thorny” issues are fundamentally essential to success of any interventions.^36, 37^ Additionally, while many technology-related questions emerged, IT solutions have been challenging to implement in the current environment because of several sociotechnical factors including poor software usability.^17^ Many of these longer term factors important to make changes in clinical practice are not reflected in our research priorities. Addressing both the complex nature of human cognitive processes and the deeply rooted systemic factors influencing the diagnostic process is essential long-term research and implementation priorities.^38^

Our study strengths include representation of a diverse group of international researchers from a variety of disciplines and use of systematic methods. Involving a large number of researchers from a variety of societies and disciplines limits the potential of personal biases to influence the outcome. We were able to address all three of the large domains previously identified as foundational to advance the field.^14^

Our limitations include a modest initial response rate, which could likely be due to otherwise busy researchers being asked to commit time to respond in absence of specific incentives. Developing and rating research questions is a time-consuming task with high cognitive workload, which affects response rates. Response rates for the CHNRI method typically vary between 30 and 70% for both researchers and stakeholders;^25^ thus, the response rates are comparable to other studies using this method. The response rate for the final prioritization exercise was much higher, reflecting an engaged group of researchers. Researchers could have been disinclined to reveal their best research questions. However, we did not witness this and in fact witnessed instances in our expert meeting where researchers advocated for their questions to be included in the priority list.

We also walked a fine line between including a broad group of participants while simultaneously ensuring sufficient expertise. Some invited participants from “outside” the research field of diagnostic safety declined participation because they felt they did not have enough knowledge to identify or evaluate research priorities in diagnostic safety. Lastly, our questions may not be fully representative. For instance, while we received input from at least 10 different countries, this may not be representative of research priorities worldwide. We included a patient representative but did not invite research questions from patient groups. We also did not delve deeper into discipline-specific research areas (e.g., lab, radiology) or how to develop capacity and a cadre of researchers to answer these research questions. Given the recent funding momentum in the field,^23, 39^ a new federal inter-agency task force on diagnostic safety,^40^ and more disciplines expressing high interest in diagnosis, these areas will be developed in due course.

In conclusion, the study identified the top short-term research priorities for advancing diagnostic safety that would be useful to both researchers and funders interested in reducing diagnostic error. Priorities broadly included addressing systems, teams, and patient engagement to support diagnosis and serve as a foundation to improve diagnostic safety and reduce preventable diagnostic harm in the near term.

## Supplementary Information

ESM 1 (DOCX 32.8 kb)
