# Supplementary material for: Advancing Diagnostic Safety Research: Results of a Systematic Research Priority Setting Exercise
Source: J Gen Intern Med. 2021 Feb 9;36(10):2943–51. doi: 10.1007/s11606-020-06428-3 (PMC8481519; doi:10.1007/s11606-020-06428-3)
Supplement: Supplementary file 1 — (DOCX 32.8 kb) [file 11606_2020_6428_MOESM1_ESM.docx]

Expert meeting attendee list, including full name, titles, area of expertise and country of residence.

| **Core Group** | **Titles** | **Area of expertise** | **Country** |
| --- | --- | --- | --- |
| Laura Zwaan | PhD | Cognitive Psychology, Medical Education | The Netherlands |
| Hardeep Singh | MD, MPH | Health Services Research | United States of America |
| Robert El-Kareh | MD, MPH, MS | Health IT, Health Services Research | United States of America |
| Ashley N.D. Meyer | PhD | Cognitive Psychology | United States of America |
|  |  |  |  |
| **Expert Panel** | **Titles** | **Area of expertise** | **Country** |
| Jeffrey Brady | MD, MPH | Funder of Quality & Patient Safety Research | United States of America |
| Ken R. Catchpole | PhD | Human Factors | United States of America |
| David A. Cook | MD, MHPE | Medical Education | United States of America |
| John T. James | PhD | Patient Safety Advocate | United States of America |
| Alan J. Forster | MD, FRCPC, MSc | Patient Safety & Quality Improvement | Canada |
| Mark Friedberg | MD, MPP | Performance Measurement & Improvement | United States of America |
| Tejal K. Gandhi | MD, MPH, CPPS | Patient and Workforce Safety | United States of America |
| Mark L. Graber | MD, FACP | Diagnostic Safety Research | United States of America |
| Ashwin B. Gupta | MD | Diagnostic Safety in Hospitalized Patients | United States of America |
| Janice L. Kwan | MD, MPH | Health Services Research | Canada |
| Michael Laposata | MD, PhD | Laboratory Medicine | United States of America |
| Prashant Mahajan | MD, MPH, MBA | Pediatric Emergency Medicine, Public Health | United States of America |
| Kathryn M. McDonald | PhD | Healthcare Quality & Patient Safety | United States of America |
| Jane K. O'Hara | PhD | Patient Safety & Improvement Science | United Kingdom |
| Emily S. Patterson | PhD | Human Factors | United States of America |
| Dean F. Sittig | PhD | Medical Informatics | United States of America |
| Daniel Yang | MD | Funder of Diagnostic Safety Research | United States of America |
